# Assessing the quality of care for skin malignant melanoma on a global, regional, and national scale: a systematic analysis of the global burden of disease study from 1990 to 2019

**DOI:** 10.1007/s00403-023-02730-2

**Published:** 2023-09-29

**Authors:** Mingjuan Liu, Mengyin Wu, Xintong Liu, Jia Zhou, Yining Lan, Hanlin Zhang, Xinyi zhang, Ling Leng, Heyi Zheng, Jun Li

**Affiliations:** 1 Department of Dermatology, Peking Union Medical College Hospital, Chinese Academy of Medical Sciences, Peking Union Medical College, Beijing, 100730 China; 2 State Key Laboratory of Complex Severe and Rare Diseases, Peking Union Medical College Hospital, Chinese Academy of Medical Sciences, Peking Union Medical College, Beijing, 100730 China; 3 4+4 M.D. Program, Chinese Academy of Medical Sciences, Peking Union Medical College, Beijing, 100005 China; 4 Peking University Health Science Center, Beijing, 100191 China; 5 Departments of Internal Medicine and Cellular and Molecular Physiology, Yale School of Medicine, New Haven, CT USA

**Keywords:** Malignant melanoma, Global burden of disease, Quality of care

## Abstract

Malignant melanoma (MM) is a highly aggressive form of skin cancer with increasing global incidence rates, particularly in developed countries. Variations in the prevalence and quality of care provided to patients with melanoma exist across different regions and across different sex and age. Assessing the global burden of melanoma and evaluating the quality of care can provide valuable insights for developing targeted interventions in certain underperforming regions and improving patient outcomes. This study aimed to systematically analyze the Global Burden of Disease Study from 1990 to 2019 to assess the quality of care for skin malignant melanoma on a global scale. We conducted a comprehensive literature review and extracted data on melanoma incidence, mortality, and disability-adjusted life years (DALYs) from the Global Burden of Disease Study. We incorporated these variables using principal component analysis (PCA) to form an informative single variable of quality of care index (QCI) and analyzed its spatial–temporal variations as well as disparities across age, sex and socio-demographic index (SDI). The overall Quality of Care Index (QCI) for melanoma improved from 82.81 in 1990 to 91.29 in 2019. The QCI score showed a positive correlation with socioeconomic status across regions. Australia ranked highest in QCI (99.96), while Central African Republic, and Kiribati had the lowest scores. China and Saudi Arabia showed significant QCI improvement, while the QCI of the Democratic People's Republic of Korea, Zimbabwe, and Guam decreased from 1990 to 2019. The highest QCI scores were observed in the age groups of 20–39 years old (93.40–94.65). Gender disparities narrowed globally in these three decades, but lower Socio-demographic Index (SDI) regions showed increased gender inequities. Our findings highlighted the spatial–temporal variations in the quality of care of MM as well as its disparities across different SDI levels, age groups and sex. These findings offer valuable insights and guidance for implementing focused interventions and resource allocation to enhance the quality of care and overall outcomes for MM worldwide, especially for underperforming regions.

**Supplementary Information:**

The online version contains supplementary material available at 10.1007/s00403-023-02730-2.

## Introduction

Cutaneous melanoma results from malignant mutation of melanocytes, the melanin-producing cells from the basal layer of the epidermis. Although melanoma only constitutes 4% of all cutaneous cancer, this malignancy leads to over 80% of skin cancer-related mortality due to its invasive potential of metastasis. The incidence of melanoma continuously rose in the past decades in developed, primarily fair-skinned countries [1]. In the United States, the rise in melanoma incidence has occurred faster than any other tumor to represent the fifth most common cancer diagnosis [2, 3]. While the likelihood of developing melanoma typically rises with advancing age, this condition is also a prominent contributor to cancer among the young, and its increasing occurrence presents a significant concern for public health burden, especially regarding years of life lost by affected individuals [4]. This pattern further underscores the importance of ensuring optimal care for MM patients.

Significant variations in the incidence of melanoma have been observed across different countries and regions worldwide. The highest rates of occurrence are found among populations with fair skin, specifically in Oceania, Western and Northern Europe, and North America. Conversely, melanoma remains relatively rare in the majority of regions in Africa, South and Central America, and Asia. However, there has reported a shift in the burden of melanoma in recent years toward the transitioning regions [5]. Some Latin American countries, such as Colombia and Mexico, have reported a significant increase in melanoma cases in recent years compared to 1990 [6, 7]. Factors contributing to the rising prevalence may comprise the lengthening of human lifespan, insufficient prevention and early detection initiatives, strained national healthcare systems, and inadequate supporting systems for clinical research [8]. In addition, developing countries, specifically in Asia and Africa, experience a higher fatality rate associated with melanoma. While Asia accounted for only 7.3% of all observed cases, a disproportionately high 21.0% of all melanoma-related deaths were reported from this region. In comparison, Oceania accounted for 5.9% of all melanoma cases, and the global deaths attributed to Oceania were only 3.4% [8].

An important challenge facing modern healthcare systems is the unequal division of quality of cancer care. A cohort study conducted by Zheng et al. using the SEER database affirmed that within the U.S., Asians and Pacific Islanders have a significantly lower risk of developing melanoma but poorer overall survival and disease-specific survival rates for melanoma as compared to Fitzpatrick type I skin types, primarily due to delayed diagnosis and treatment [9]. The World Health Organization (WHO) defines quality of care (QoC) as the provision of healthcare services that improve desired health outcomes for individuals and populations through effective, efficient, and safe means [10]. However, the quality of care for MM on a national, regional and global level remained unclear. Given the significant differences in melanoma incidence and mortality and the limited literature on its quality of care in developing countries, there is a need to comprehensively investigate the quality of care index (QCI) specific to melanoma that across different world regions, genders and ages. This comprehensive and comparative study of QCI has the potential to provide experts and policymakers with a worldwide visualization of the current melanoma quality of care status and raise public health awareness, leading to possible interventions and strategies to resolve existing inequalities in healthcare access and improve the quality of care in regions with low-quality care in the future.

## Methods

### Data source

We utilized the GBD Study 2019 online Global Health Data Exchange query tool to gather data on melanoma worldwide [11]. For assessing the melanoma-related burden, GBD encompassed its prevalence, incidence, mortality, DALYs (Disability-Adjusted Life Years), and age-standardized rates from multiple national cancer registry systems and aggregated databases. Melanoma is identified under diagnostic ICD-10 codes (C43-C43.9, Z85.82-Z85.828) and ICD-9 codes (172–172.9) in this study. We conducted data collection and analysis of melanoma QCI at global, regional, and national levels encompassing primary indices of prevalence, incidence, mortality, years of life lost (YLL), and years lost due to disability (YLD) and DALY data from 1990 to 2019. This study adheres to the Guidelines for Accurate and Transparent Health Estimates Reporting (GATHER) statements.

### QCI derivation

The QCI should be a comprehensive index that represents the overall quality of care for melanoma. To calculate the Quality of Care Index (QCI) for melanoma, we incorporated several primary and secondary indices derived from GBD 2019 and employed principal component analysis. This derivation of QCI was first introduced by Mohammadi et al. and has been validated in their QCI analysis for several non-communicable diseases including cancers [12–15]. The four secondary indices for PCA analysis of QCI consist of

- Mortality to incidence ratio (MIR): Calculated as the ratio of mortality to incidence, indicating the relative mortality rate for a fixed incidence rate. This ratio was confirmed as a valid measure for assessing quality of care and healthcare performance in various types of cancer [16].
- Prevalence to incidence ratio (PIR): Calculated as the ratio of prevalence to incidence, indicating the relative prevalence and potential for prevention. Higher PIR suggests a decrease in melanoma incidence and potentially improved preventative programs.
- YLL to YLD ratio: Calculated as the ratio of YLLs to YLDs, indicating the proportion of years lost due to premature death compared to years lived with disability, for which higher values indicate more deaths than disabilities caused by MM. This index underscores the effectiveness of health systems in delaying patients’ death.
- DALY to prevalence ratio: calculated as DALYs (including both YLL and YLD) divided by prevalence, indicating the burden of melanoma for a fixed prevalence rate.

The first principal component extracted from PCA represents a linear combination of these secondary indices that explains the most variation, captures the most significant information about the quality of care and is therefore scaled from 0 to 100 to represent QCI for melanoma. QCI is calculated for different geographic regions, such as countries, WHO regions, and GBD regions over the 1990–2019 period for different age groups and genders.

### QCI validation

We sought to validate the quality of care index (QCI) by examining its correlation with the healthcare access and quality (HAQ) index previously developed by IHME, which has served as a recognized measure of care quality and access [17]. We utilized a mixed-effects model where QCI was treated as the dependent variable. The independent variables included outpatient care utilization, inpatient care utilization, MM prevalence and deaths. Countries were considered as random effects in the model to account for potential variations between countries. We observed a Pearson’s correlation coefficient of 0.90 between the predicted QCI values and HAQ index, indicating a strong positive association between the two indices.

### The pattern of disparities

#### Socio-Demographic index (SDI)

SDI was employed to categorize countries according to their socioeconomic development. SDI is a comprehensive measure that incorporates average income per person, educational attainment, and total fertility rate [18]. The 204 countries and territories included in the global burden of disease (GBD) study were classified into five SDI quintiles: high, high-middle, middle, low-middle, and low. These predefined thresholds enabled a systematic evaluation of the countries' socioeconomic status and facilitated comparative analysis.

#### Gender disparities

The quality of care was assessed separately for women and men by calculating age-standardized QCI scores. The gender disparity ratio (GDR) was obtained by dividing the QCI scores of females by the scores of males. A GDR value closer to one indicated more equitable outcomes and a lesser disparity in quality of care between genders. Values above one represented better quality of care for females, while values below one indicated better quality of care for males, indicating potential gender disparities. By utilizing GDR, an investigation was conducted into gender inequities in the provision of care for melanoma patients, aiming to identify areas for improvement from gender inequalities.

#### Age disparities

Age disparity was examined to understand the disparities in quality of care across different age groups within SDI. Most age groups are classified according to a 5-year age interval. This analysis provided insights into the variations in the quality of care experienced by different age cohorts, allowing for a comprehensive understanding of age-related disparities in melanoma care.

### Statistical analysis

The QCI values for various regions, countries, age groups, and sex groups were computed using the entire GBD dataset for melanoma, which contained no missing data for any entries. Primary indices were accompanied by a 95% uncertainty interval (UI). The Quality of Care Index was calculated using principal component analysis as discussed previously. Pearson's correlation analysis was used to examine the correlation between SDI and QCI, and a significance level of *P* < 0.05 was employed. All statistical analyses were conducted using R statistical packages v 4.2.0. For data visualizations, we utilized the ‘ggplot2’ package from R studio. The statistical codes utilized in this study are accessible from previous literature [19].

## Results

### Overview

On a global level, the age-standardized DALYs for MM were estimated to be 23.58 (19.62–29.92) in 1990 and decreased to 20.81 (15.78–24.33) in 2019. Similarly, the mortality rate for MM was estimated to decrease from 0.85 (0.72–1.10) in 1990 to 0.79 (0.58–0.89) in 2019. The overall Quality of Care Index (QCI) for MM was 82.81 in 1990 and improved to 91.29 in 2019 (Table 1).

**Table 1:** The disability-adjusted life years (DALYs) and mortality rates and quality of care index (QCI) in 1990 and in 2019 for different regions, sex and age categories

Location	Sex	Age	1990			2019		
			DALYs^a^	Deaths	QCI^b^	DALYs^a^	Deaths	QCI^b^
Global	Both	Age-standardized	23.58 (19.62–29.92)	0.85 (0.72–1.10)	82.81	20.81 (15.78–24.33)	0.79 (0.58–0.89)	91.29
	Both	All ages	19.17 (15.94–24.28)	0.62 (0.52–0.81)	84.60	22.07 (16.74–25.82)	0.81 (0.60–0.92)	92.17
	Female	Age-standardized	20.68 (17.43–28.75)	0.73 (0.61–1.03)	84.96	17.28 (12.43–20.80)	0.63 (0.44–0.74)	92.20
	Male	Age-standardized	26.88 (19.37–36.32)	0.99 (0.73–1.39)	80.81	24.83 (16.21–30.76)	0.98 (0.60–1.17)	90.50
High SDI	Both	Age-standardized	50.60 (37.73–62.56)	1.64 (1.26–2.16)	92.38	48.06 (34.82–59.93)	1.63 (1.12–1.93)	97.94
		All ages	59.41 (44.30–74.22)	2.01 (1.55–2.66)	92.45	73.34 (51.45–89.46)	2.90 (1.92–3.38)	97.24
High-middle SDI	Both	Age-standardized	26.97 (22.65–35.02)	0.93 (0.80–1.25)	72.02	25.70 (18.81–29.57)	0.91 (0.66–1.03)	88.76
		All ages	26.26 (21.98–34.04)	0.85 (0.72–1.14)	73.15	34.31 (24.94–39.07)	1.26 (0.90–1.42)	88.71
Middle SDI	Both	Age-standardized	10.04 (7.69–13.55)	0.37 (0.29–0.48)	32.63	9.51 (7.54–11.33)	0.37 (0.29–0.44)	69.53
		All ages	7.39 (5.62–10.12)	0.22 (0.17–0.29)	35.83	10.16 (8.00–12.14)	0.36 (0.28–0.43)	71.03
Low-middle SDI	Both	Age-standardized	8.07 (5.67–11.82)	0.29 (0.22–0.41)	19.09	8.52 (6.63–10.26)	0.31 (0.24–0.37)	46.59
		All ages	5.46 (3.73–8.23)	0.16 (0.11–0.23)	20.98	7.42 (5.76–8.91)	0.24 (0.18–0.28)	48.49
Low SDI	Both	Age-standardized	14.18 (9.40–21.77)	0.48 (0.34–0.69)	9.55	13.79 (10.24–17.49)	0.47 (0.35–0.59)	24.10
		All ages	8.77 (5.49–14.69)	0.24 (0.16–0.37)	10.59	8.73 (6.56–11.27)	0.24 (0.18–0.30)	27.45
Andean Latin America	Both	Age-standardized	22.71 (17.32–32.69)	0.89 (0.69–1.24)	23.35	20.56 (14.69–29.23)	0.86 (0.61–1.15)	58.69
Australasia	Both	Age-standardized	138.36 (104.35–182.31)	4.41 (3.42–6.06)	97.17	128.92 (93.12–169.58)	4.37 (2.96–5.41)	99.83
Caribbean	Both	Age-standardized	13.36 (11.18–18.79)	0.48 (0.41–0.66)	55.59	14.26 (11.26–19.14)	0.52 (0.42–0.69)	71.61
Central Asia	Both	Age-standardized	19.93 (14.53–23.16)	0.76 (0.53–0.88)	50.86	17.83 (15.09–24.97)	0.73 (0.62–1.00)	65.45
Central Europe	Both	Age-standardized	53.32 (44.47–69.79)	1.76 (1.51–2.37)	68.77	59.98 (43.25–73.01)	2.09 (1.47–2.52)	88.05
Central Latin America	Both	Age-standardized	14.34 (12.63–21.36)	0.55 (0.47–0.80)	41.77	18.16 (14.10–25.22)	0.71 (0.54–0.96)	69.76
Central Sub-Saharan Africa	Both	Age-standardized	17.11 (11.51–26.57)	0.63 (0.44–0.88)	8.03	16.63 (11.84–23.56)	0.63 (0.44–0.86)	17.40
East Asia	Both	Age-standardized	9.52 (6.50–13.93)	0.33 (0.24–0.48)	33.73	8.08 (5.27–10.05)	0.28 (0.19–0.35)	84.65
Eastern Europe	Both	Age-standardized	37.75 (30.06–52.09)	1.19 (0.99–1.74)	69.59	54.83 (41.95–66.45)	1.70 (1.28–2.06)	86.08
Eastern Sub-Saharan Africa	Both	Age-standardized	23.98 (16.12–37.68)	0.77 (0.54–1.16)	10.16	23.36 (17.22–31.48)	0.78 (0.57–1.03)	24.47
High-income Asia Pacific	Both	Age-standardized	6.78 (6.06–9.16)	0.24 (0.21–0.33)	87.91	7.07 (4.79–8.27)	0.24 (0.16–0.27)	98.01
High-income North America	Both	Age-standardized	72.36 (50.75–86.30)	2.25 (1.65–2.81)	94.11	64.24 (51.69–89.70)	2.20 (1.63–2.85)	97.67
North Africa and Middle East	Both	Age-standardized	12.14 (6.93–17.66)	0.47 (0.28–0.65)	36.21	9.58 (6.38–11.64)	0.40 (0.26–0.48)	72.03
Oceania	Both	Age-standardized	12.49 (8.49–22.01)	0.49 (0.34–0.84)	9.14	12.13 (8.08–19.37)	0.48 (0.34–0.75)	9.95
South Asia	Both	Age-standardized	5.26 (3.80–7.50)	0.20 (0.15–0.27)	15.93	5.38 (3.85–6.50)	0.19 (0.14–0.24)	41.34
Southeast Asia	Both	Age-standardized	6.90 (5.40–10.48)	0.25 (0.20–0.36)	13.45	6.39 (5.14–8.83)	0.25 (0.20–0.34)	29.93
Southern Latin America	Both	Age-standardized	27.90 (24.02–41.48)	0.99 (0.85–1.48)	55.17	35.44 (25.61–45.44)	1.28 (0.91–1.60)	78.65
Southern Sub-Saharan Africa	Both	Age-standardized	30.55 (23.39–39.96)	1.13 (0.84–1.42)	27.39	32.67 (21.66–39.86)	1.27 (0.80–1.53)	37.90
Tropical Latin America	Both	Age-standardized	27.87 (21.78–38.32)	0.97 (0.75–1.32)	41.16	28.61 (23.88–41.90)	1.05 (0.82–1.47)	66.81
Western Europe	Both	Age-standardized	51.79 (39.94–67.67)	1.67 (1.33–2.26)	90.41	57.40 (35.15–65.13)	1.89 (1.13–2.10)	98.28
Western Sub-Saharan Africa	Both	Age-standardized	11.26 (7.20–15.57)	0.40 (0.27–0.53)	13.29	10.85 (7.38–13.83)	0.40 (0.27–0.50)	26.98

^a^*DALYs* disability-adjusted life years. Data in parentheses are 95% uncertainty intervals

^b^*QCI* quality of care index

However, the analysis also revealed that the all-age DALYs for MM increased from 19.17 (15.94–24.28) in 1990 to 22.07 (16.74–25.82) in 2019, suggesting a notable rise in the overall burden of MM on the global population during the studied period. Similarly, the all-age mortality rate for MM showed an upward trend, increasing from 0.62 (0.52–0.81) in 1990 to 0.81 (0.60–0.92) in 2019.

When examining the data by gender, both males and females showed improvements in the age-standardized DALYs, mortality rates, and QCI over the studied period. In 2019, males had a higher age-standardized DALY value of 24.83 (16.21–30.76) compared to females with 17.28 (12.43–20.80). Similarly, the mortality rate for males was 0.98 (0.73–1.39), while 0.63 (0.44–0.74) for females.

### QCI

The analysis revealed huge disparities in the quality of care for melanoma patients across GBD regions. In 2019, Australasia had the highest average QCI score (99.83) among WHO regions, followed closely by Western Europe (98.28) and High-income Asia Pacific (98.01). On the other hand, Oceania and Central Sub-Saharan Africa had the lowest average QCI (9.95 and 17.40 respectively), indicating the need for targeted interventions and resource allocation to improve care quality in underperforming areas.

**Fig. 1:**
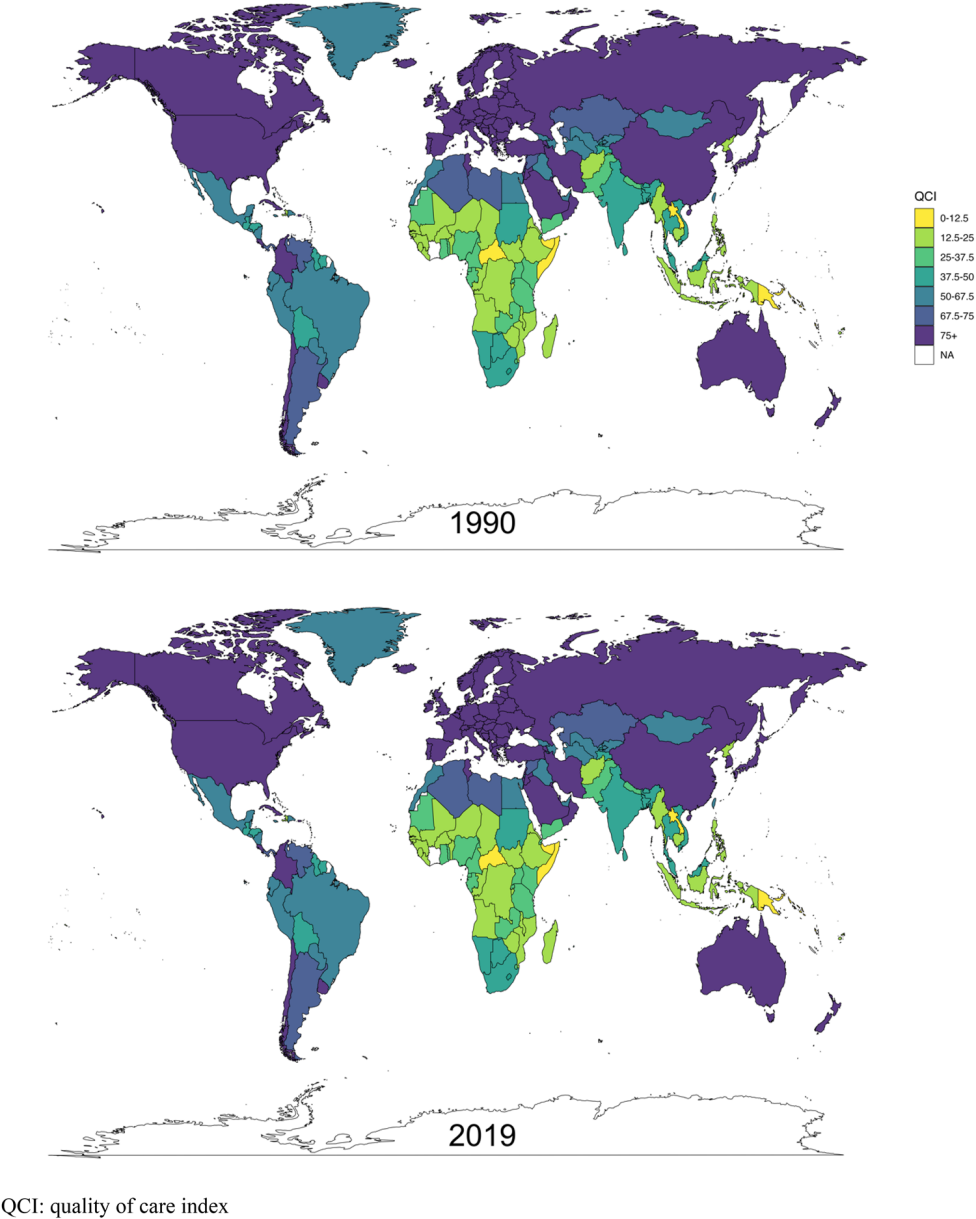
Age-standardized quality of care index (QCI) for melanoma by country in 1990 and 2019

The highest-ranked country in terms of quality of care in 2019 was Australia with a QCI score of 99.96. Other countries that consistently performed well in the studied period include Andorra, Netherlands, New Zealand and Monaco (Fig. 1). On the other end of the spectrum, the Central African Republic, Kiribati and Somalia ranked among the lowest-performing countries in 2019 with QCI scores below 6.00. Notably, these countries had also been among the lowest QCI-ranked nations in 1990, indicating the persistent and enduring challenges they faced in providing sufficient care for MM patients (Fig. 1).

When analyzing the countries with notable improvements in the quality of care for MM, China and Saudi Arabia emerge as standout countries with their remarkable increase in QCI scores increase by 51.64 and 46.44 over the three decades. Conversely, the Democratic People's Republic of Korea, Zimbabwe, and Guam experienced some decrease in QCI over time.

### Age and gender disparities

**Fig. 2:**
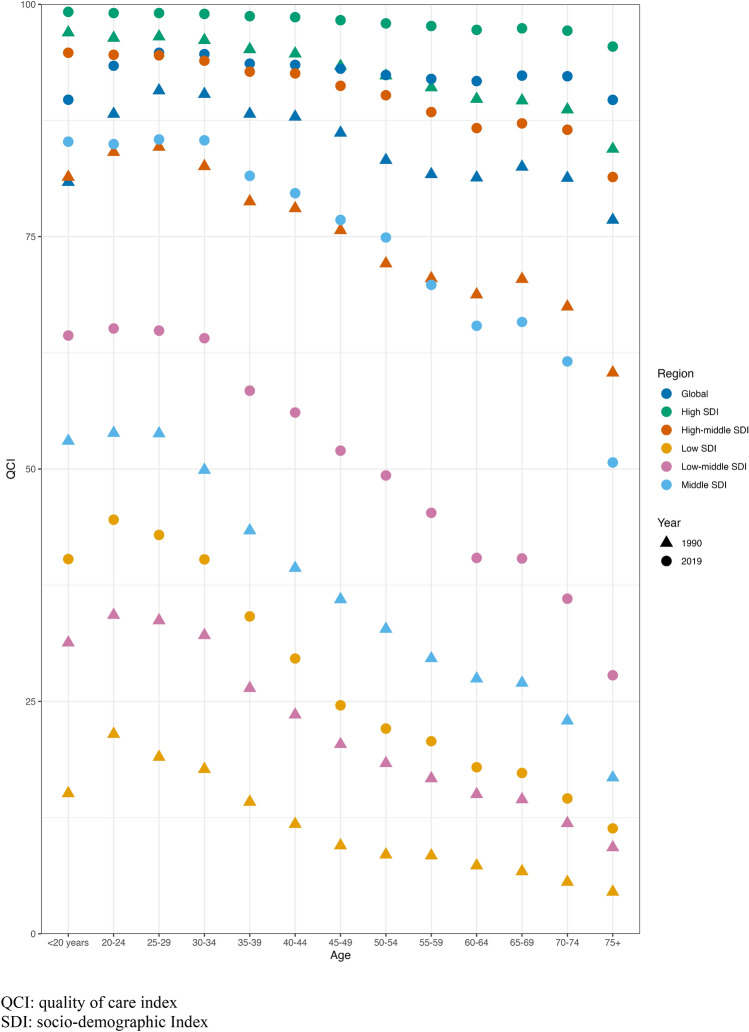
Quality of care index (QCI) among people of different age groups in 1990 and in 2019

Overall QCI continually decreases as age advances from 25 to 29 years age group (Fig. 2), with a less noticeable disparity in high Socio-demographic Index (SDI) regions but a more prominent decline in lower SDI regions (from 65.10 to 27.80). Globally, the age groups ranging from 20 to 39 years old exhibited the highest quality of care in 2019, with QCI scores ranging from 93.40 to 94.65 (Fig. 2).

**Fig. 3:**
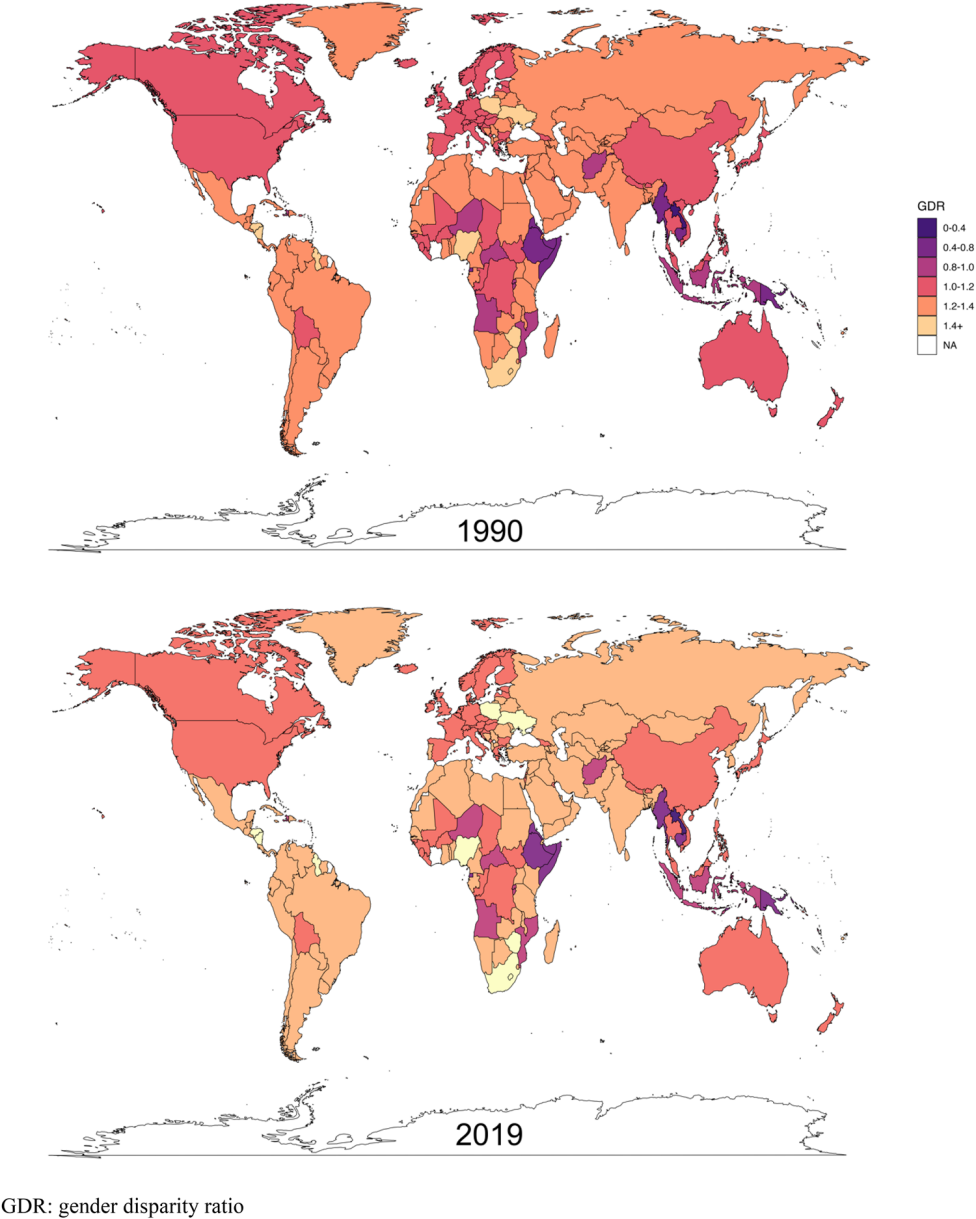
Age-standardized gender disparity ratio (GDR) for melanoma by country in 1990 and 2019

The global GDR score was 1.05 in 1990 and 1.01 in 2019, suggesting an overall similar quality of care for female and male patients. As shown in Fig. 3, The GDR scores decreased in most areas from favoring women in 1990 to approaching more gender equality (closer to 1.00) in 2019, as observed in 144 countries (70.6% of the total). In 2019, all SDI quintiles showed a better quality of care for women compared to men, despite higher SDI quintiles exhibiting minimal inequities: the GDR was 1.01 for high SDI quintiles, 1.06 for high-middle, 1.10 for middle, 1.19 for low-middle, and 1.41 for low SDI quintiles. Despite the global trend of narrowing gender disparities, one concerning trend can be observed in the low SDI quintile as its GDR has shown a progression towards gender inequity, increasing from 1.14 to 1.41 (Fig. S1). The GDR remained stable and close to one globally and in high SDI regions across different age groups (Fig. S1). The countries with the highest gender disparity favoring women were Comoros (GDR = 1.49), Zambia (GDR = 1.49), and Rwanda (GDR = 1.48). Conversely, only the Central African Republic, Somalia, Kiribati, Papua New Guinea, and China had a GDR lower than 1, revealing a male advantage in melanoma care quality.

### Temporal and spatial variations in QCI

**Fig. 4:**
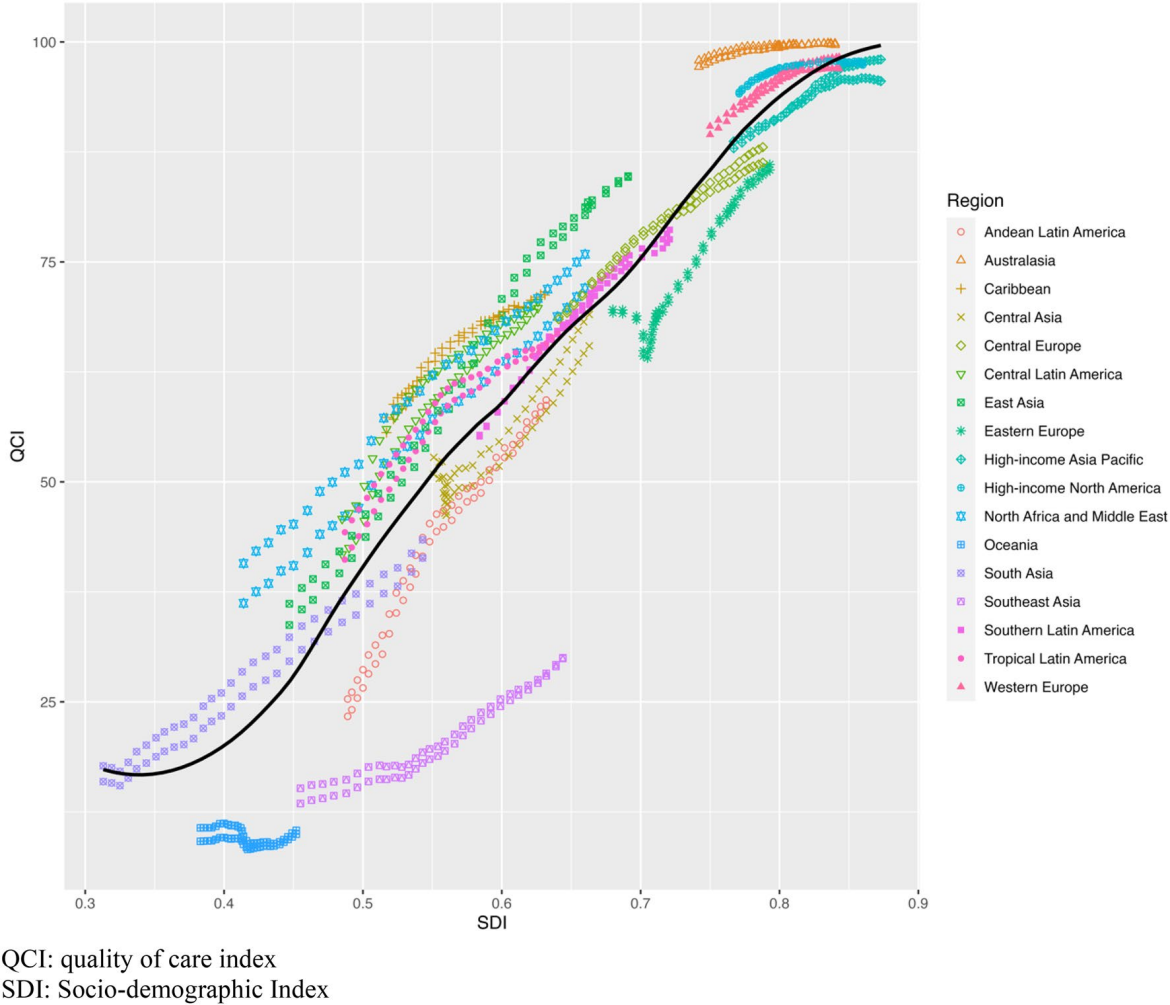
The temporal trend in the quality of care index (QCI) from 1990 to 2019 and its positive correlation with the Socio-demographic Index (SDI) in 21 WHO regions. Expected values are shown as the dark line

Figure 4 illustrates the overall consistent improvement of the QCI from 1990 to 2019 in all World Health Organization regions, except for in Eastern Europe, which encountered remarkable dips in QCI during the mid-1990s. The QCI score demonstrates a positive correlation with the socio-economic status across WHO regions and countries (Pearson’s correlation *r* = 0.86; 95% CI 0.85–0.86; *P* < 0.001), as also depicted in Fig. 4, Figs. S2 and S3.

## Discussion

This study aimed to conduct a comprehensive evaluation of the quality of care and disparities associated with malignant melanoma (MM) on global, national and regional scales using the validated quality of care index. The analysis of data from the Global Burden of Disease Study (GBD) 2019 revealed both positive and concerning trends pertaining to the burden and quality of care for MM. On a positive note, a declining trend in the age-standardized disability-adjusted life years and mortality rates was observed for MM between the years 1990 and 2019. However, despite these improvements, there was a concerning upward trend observed in the all-age mortality and DALYs for MM over the same period, suggesting an increased overall burden of MM. The rationale behind this divergent trend may be attributed to improved life expectancy and the demographic shift towards population aging, alongside potential contributions from enhanced awareness, surveillance, and earlier detection practices.

The gender disparity in the quality of care of MM was estimated to be modest on a global level. Nevertheless, a notable finding of the study was that female MM patients generally have a better quality of care compared to their male counterparts in most countries. One primary explanation for this observation is the fact that MM is frequently diagnosed at an earlier stage in women. In general, females often exhibit a greater emphasis on maintaining skin health and on regular skin examinations, which enables the detection of melanomas at a younger age, facilitating earlier intervention and more effective treatment outcomes [20, 21]. In comparison, males typically possess less knowledge regarding skin health and are less likely to engage in self-checks or seek regular visits to dermatologists [22]. The discrepancies are further described in diagnostic delays in males as manifested in their increased Breslow thickness, older age and higher AJCC stage at initial diagnosis [22, 23]. However, better survival outcomes were consistently observed in females even after adjusting for the factors including diagnosis delays, age, and Breslow thickness [24–26], arguing for an intrinsic biological sex advantage in women. A large-cohort study of 10,538 MM patients conducted by de Vries et al. revealed that men overall had a worse relative excess risk (RER) of mortality as compared to women (RER 2.70, 95% CI 2.38–3.06) [25]. Several hypotheses have been proposed regarding biological sex differences in relation to melanoma, such as the impact of estrogen receptor expression [27] and variances in the ability to neutralize oxidative stress [28, 29]. Exploring the biological factors that underlie gender differences in melanoma prognosis could have important implications as it may provide insights into the mechanisms driving divergent melanoma survival outcomes and be applied in prognosis evaluation and even in potentially devising targeted therapy. For public health, these findings also emphasize the need for targeted programs to address the specific challenges faced by men in preventing, detecting and managing melanoma.

The positive correlation between SDI and QCI in the analysis aligns with expectations because of the availability of early diagnosis, high-quality care, screening and preventative programs, and the concentration of scientific efforts in countries with higher SDI to improve melanoma outcomes. Conversely, individuals in low SDI countries may face challenges in accessing optimal care for melanoma. Additionally, in many low SDI regions, healthcare priorities may need to focus more on communicable diseases due to their high prevalence and disease burden (such as malaria, HIV/AIDS, tuberculosis, and various neglected tropical diseases) [30], which could impact the allocation of resources and attention given to cancer care. However, there was a consistent gradual increase in QCI in regions of lower SDI, but minimal improvement was observed in the QCI for the high SDI group for the last ten years. Despite the quickest access to advanced therapies in high SDI countries, this stagnant QCI status suggests that scientific advancements in melanoma in recent years have not significantly improve the overall quality of care for melanoma patients in high SDI region yet.

Countries with favorable QCI scores can offer valuable insights for policymakers seeking to improve the quality of care for melanoma through the implementation of preventative and educational programs. Australia, as ‘the sunburnt country’, has implemented multiple effective skin cancer programs, such as the renowned ‘SunSmart’ program that emphasized sun protection measures, such as wearing protective clothing, using sunscreen, seeking shade, and undergoing regular skin checks [31, 32]. Mass media resources were applied to raise awareness and promote preventative behaviors, targeting both the general population and specific high-risk groups, such as teenagers and outdoor workers [33]. The messages conveyed by the campaign are further reinforced by comprehensive social supportive systems, including the availability of outdoor shading and access to quality sun-protection products. Early detection has also been a key focus, with efforts directed toward improving skin cancer detection and diagnosis among general practitioners, as well as establishing accessible skin cancer clinics throughout the country [33]. Ultimately, achieving high quality of care for MM requires a multi-faceted approach that encompasses healthcare infrastructure improvement, education, research, and advocacy for affordable and accessible healthcare services.

Another example of a multicomponent melanoma campaign is Euromelanoma, with over 85% of the participating countries achieving high QCI or remarkable improvements. This pan-European campaign prioritizes the prevention, risk-factor identification and early diagnosis of skin cancers [34]. Annual free skin screenings are promoted, and standardized questionnaires were used for data on skin cancer demographics and risk factors, which have informed concrete legislative actions against tanning beds [34, 35]. Additionally, its comprehensive online resources have been informing about skin cancer prevention and risk factors, as well as the self-examinations of the skin to the European public [36]. Tailoring the message to address a high-risk population has proven effective in Belgium and Sweden [37]. In Belgium, the 2007 Euromelanoma campaign targeted men over fifty, a demographic at elevated risk for melanoma mortality. A former Belgian prime minister lent his support by sharing his body image, after which this proportion of male attendance increased from 37% to 64% [38]. Building on its prior successes, Euromelanoma's future direction lies in the further identification and understanding of melanoma risk factors across different populations [34].

Peru is another example of remarkable progress in melanoma healthcare, with a QCI increase from 27.14 to 65.74. The melanoma campaign in Peru is characterized by a strong legislative approach since 2003 [39]. This involves regulations to safeguard schoolchildren by limiting activities with unprotected UVR exposure, overseeing school uniforms, and ensuring UVR exposure risk education in the curriculum. Employers are mandated to furnish sunscreens and suitable protective attire for UVR-exposed employees. Public parks are obligated to feature signs emphasizing the risks of extended UVR exposure [39]. Additionally, their annual free mole screenings have been established as an official national event, leading to the identification of melanoma in approximately 0.74% of the 118,092 participating citizens [40].

However, regarding the southern African regions covering countries with QCI below 50—like Lesotho, Namibia, Botswana, and South Africa—prior GBD research on melanoma highlighted their lack of effective skin cancer prevention campaigns [41]. As an example, while South Africa has implemented public campaigns and educational programs for melanoma prevention and self-examination, the scarcity of skin screening initiatives and the lack of clinical resources are conspicuous challenges (166 dermatologists serving a population surpassing 40 million) [42], potentially impeding advancements in melanoma healthcare. While effective campaigns in developed countries offer substantial insights to countries striving for enhanced melanoma healthcare, it is crucial to acknowledge the significant variations in the manifestations and underlying etiology of melanoma across different regions and ethnicities [43]. In Africa, the lower limbs were consistently reported as the most frequent site (over 70%) for overall cutaneous cancers, with a substantial proportion of melanoma being acral lentiginous melanoma [44–46]. This finding implied a possible reduced contribution of ultraviolet radiation to the pathogenesis of cutaneous neoplasm in this population [47]. For health campaigns promoting melanoma prevention in this region, the educational focus should shift partially from cultivating sun-seeking behaviour to regular skin self-examinations of the soles and legs, protective footwear, and potentially proper wound care.

The strengths of our study are evident in several aspects. Multiple important epidemiological indicators were utilized to constitute mortality-to-incidence ratio, DALYs-to-prevalence ratio, prevalence-to-incidence ratio and YLLs-to-YLDs ratio, which were then incorporated into the derivation of a single outcome-oriented objective index of quality of care. This approach enables a holistic assessment of the quality of care among countries and geographical and SDI regions as well as facilitates comparisons across age and sex.

There are also several limitations of this study. Our study shares the limitations of the GBD study as we relied on its primary data. For example, ethnic and racial inequalities could not be estimated due to the lack of available data in GBD 2019. Secondly, the GBD study relies on statistical modeling and predictive covariates in locations with limited data sources, and the accuracy of the primary indices used in the calculation of QCI for these regions should be treated more cautiously. Additionally, the potential impact of enhancements in data reporting between 1990 and 2019 should be interpreted with caution. The improvement in data reporting quality can confound the actual trend in QCI by mirroring a trend partially towards data collection. Lastly, although the application of principal component analysis (PCA) enabled an informative single variable of QCI, this method does not allow for the presentation of uncertainty values and limits the ability to assess the precision of the QCI estimates.

This systematic analysis of the quality of care for skin malignant melanoma highlights the significant regional disparities, age and gender disparities, and temporal trends observed worldwide. It underscores the need for targeted interventions, resource allocation, and addressing socio-economic disparities to improve care quality and outcomes for MM patients globally. Continued efforts to enhance care delivery, particularly in underperforming regions, are crucial to ensure equitable access and improve the overall quality of care for individuals affected by skin malignant melanoma. Harnessing the impacts of social media can serve as a powerful tool today to enhance message delivery and awareness and promote preventive measures, contributing to better care for the public and improved outcomes for MM patients.

### Supplementary Information

Below is the link to the electronic supplementary material.

Supplementary file1 (DOCX 234 kb)

Supplementary file2 (DOCX 384 kb)

Supplementary file3 (DOCX 372 kb)

Supplementary file4 (DOCX 42 kb)

## Data Availability

The primary data in this study for analysis can be retrieved from https://vizhub.healthdata.org/gbd-results/.
