# Supplementary material for: Assessing the quality of care for skin malignant melanoma on a global, regional, and national scale: a systematic analysis of the global burden of disease study from 1990 to 2019
Source: Arch Dermatol Res. 2023 Sep 29;315(10):2893–904. doi: 10.1007/s00403-023-02730-2 (PMC10615953; doi:10.1007/s00403-023-02730-2)
Supplement: Supplementary file 2 — Supplementary file2 (DOCX 384 kb) [file 403_2023_2730_MOESM2_ESM.docx]

**
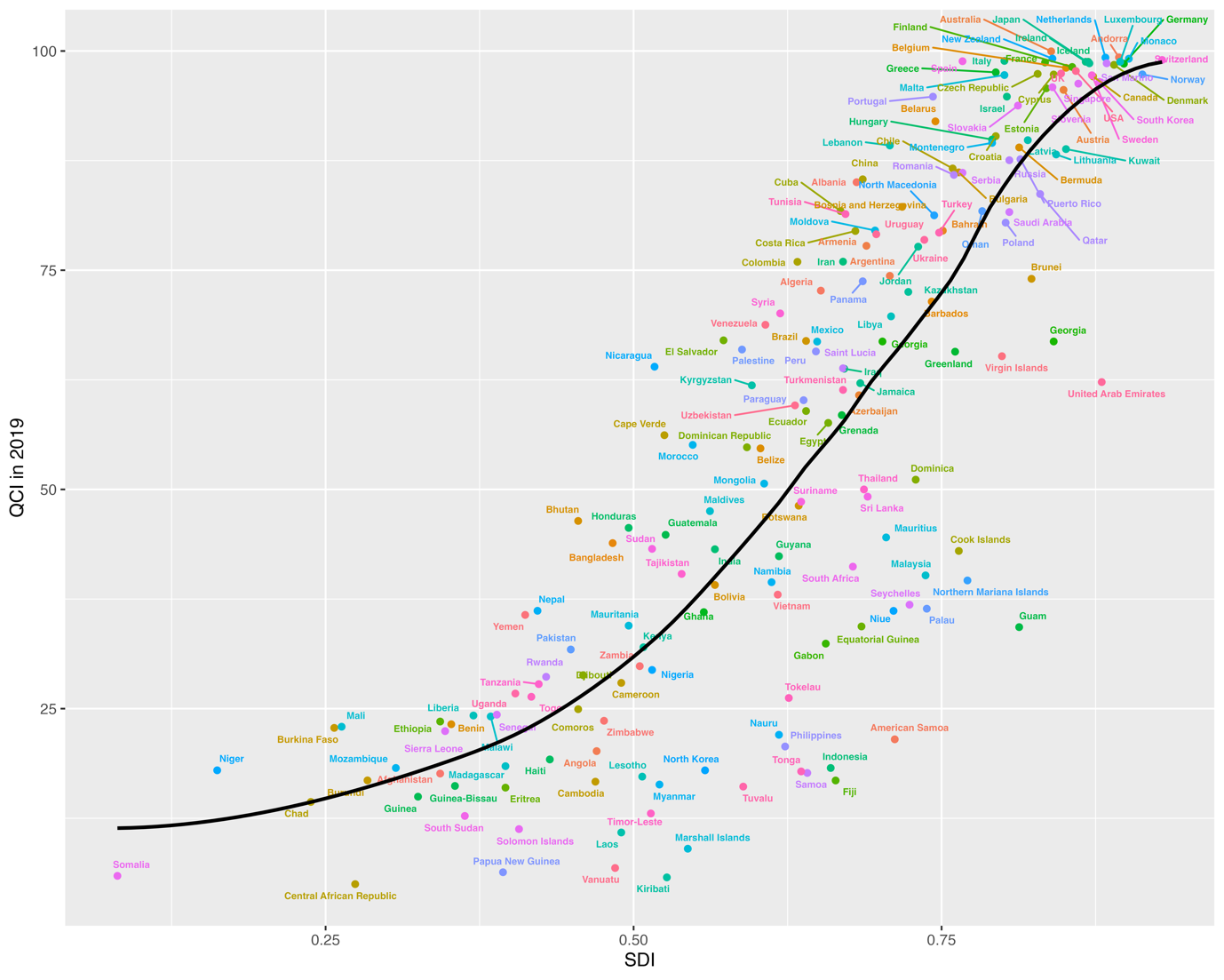
**

QCI: quality of care index

SDI: Socio-demographic Index

**Figure S2.** Age-standardized quality of care index (QCI) for malignant melanoma in 204 countries on Socio-demographic Index (SDI) scales in 2019
