# Supplementary material for: Assessing the quality of care for skin malignant melanoma on a global, regional, and national scale: a systematic analysis of the global burden of disease study from 1990 to 2019
Source: Arch Dermatol Res. 2023 Sep 29;315(10):2893–904. doi: 10.1007/s00403-023-02730-2 (PMC10615953; doi:10.1007/s00403-023-02730-2)
Supplement: Supplementary file 4 — Supplementary file4 (DOCX 42 kb) [file 403_2023_2730_MOESM4_ESM.docx]

**Table S1.**The quality of care index (QCI) in 1990, 2000, 2010 an in 2019 for 204 countries

| **Country** | **QCI*** | | | |
| --- | --- | --- | --- | --- |
|  | **1990** | **2000** | **2010** | **2019** |
| Afghanistan | 7.54 | 6.98 | 11.78 | 17.60 |
| Albania | 52.61 | 66.75 | 80.14 | 85.04 |
| Algeria | 41.84 | 51.94 | 61.49 | 72.67 |
| American Samoa | 19.43 | 20.66 | 20.12 | 21.50 |
| Andorra | 92.80 | 97.68 | 98.97 | 99.27 |
| Angola | 4.96 | 6.60 | 12.68 | 20.16 |
| Antigua and Barbuda | 53.41 | 58.59 | 65.14 | 68.33 |
| Argentina | 52.85 | 64.64 | 68.75 | 74.36 |
| Armenia | 55.76 | 61.85 | 71.33 | 77.78 |
| Australia | 97.54 | 99.31 | 99.81 | 99.96 |
| Austria | 86.81 | 92.68 | 94.22 | 95.56 |
| Azerbaijan | 40.18 | 41.26 | 51.52 | 60.74 |
| Bahamas | 49.32 | 58.76 | 61.65 | 62.53 |
| Bahrain | 43.46 | 57.17 | 71.11 | 79.51 |
| Bangladesh | 12.47 | 18.17 | 30.28 | 43.88 |
| Barbados | 55.22 | 64.50 | 70.16 | 71.42 |
| Belarus | 73.38 | 76.03 | 84.52 | 91.98 |
| Belgium | 89.61 | 94.41 | 96.89 | 98.08 |
| Belize | 39.69 | 42.46 | 52.08 | 54.68 |
| Benin | 12.67 | 14.92 | 19.97 | 23.22 |
| Bermuda | 65.27 | 79.55 | 86.18 | 89.00 |
| Bhutan | 11.79 | 20.65 | 35.45 | 46.41 |
| Bolivia (Plurinational State of) | 12.06 | 20.22 | 29.92 | 39.10 |
| Bosnia and Herzegovina | 56.40 | 68.60 | 79.08 | 82.23 |
| Botswana | 22.74 | 28.69 | 36.04 | 48.14 |
| Brazil | 41.21 | 53.62 | 61.59 | 66.94 |
| Brunei Darussalam | 55.21 | 64.82 | 70.06 | 74.02 |
| Bulgaria | 75.16 | 76.44 | 80.76 | 86.16 |
| Burkina Faso | 12.05 | 15.03 | 20.23 | 22.81 |
| Burundi | 7.04 | 8.26 | 15.35 | 16.82 |
| Cabo Verde | 32.16 | 35.04 | 47.97 | 56.18 |
| Cambodia | 3.84 | 4.96 | 10.17 | 16.67 |
| Cameroon | 13.34 | 13.16 | 18.78 | 27.94 |
| Canada | 90.64 | 94.53 | 96.51 | 97.11 |
| Central African Republic | 4.15 | 2.99 | 4.70 | 4.99 |
| Chad | 9.51 | 7.96 | 10.79 | 14.36 |
| Chile | 58.20 | 71.97 | 81.98 | 86.60 |
| China | 33.73 | 52.17 | 76.52 | 85.37 |
| Colombia | 42.56 | 58.80 | 67.96 | 75.96 |
| Comoros | 8.79 | 11.50 | 18.30 | 24.94 |
| Congo | 9.11 | 8.51 | 16.36 | 21.99 |
| Cook Islands | 32.26 | 33.93 | 37.90 | 42.98 |
| Costa Rica | 63.39 | 68.84 | 75.38 | 79.47 |
| Croatia | 74.72 | 80.52 | 87.17 | 90.29 |
| Cuba | 66.47 | 72.64 | 77.67 | 81.75 |
| Cyprus | 77.50 | 87.13 | 96.01 | 97.32 |
| Czechia | 84.47 | 93.56 | 96.11 | 97.39 |
| Democratic People's Republic of Korea | 22.32 | 12.36 | 14.75 | 17.96 |
| Democratic Republic of the Congo | 8.76 | 8.57 | 10.61 | 15.59 |
| Denmark | 89.14 | 93.95 | 97.50 | 98.42 |
| Djibouti | 12.47 | 10.75 | 19.04 | 28.82 |
| Dominica | 46.80 | 53.38 | 52.83 | 51.11 |
| Dominican Republic | 30.07 | 44.63 | 50.76 | 54.81 |
| Ecuador | 25.01 | 43.54 | 51.16 | 58.94 |
| Egypt | 26.51 | 38.09 | 46.37 | 57.59 |
| El Salvador | 31.42 | 50.81 | 60.82 | 67.00 |
| Equatorial Guinea | 3.41 | 10.76 | 26.44 | 34.37 |
| Eritrea | 2.21 | 5.76 | 9.53 | 15.99 |
| Estonia | 78.91 | 84.39 | 92.79 | 95.74 |
| Eswatini | 16.80 | 14.30 | 14.87 | 25.48 |
| Ethiopia | 3.97 | 5.82 | 13.30 | 23.53 |
| Fiji | 13.71 | 12.72 | 13.86 | 16.81 |
| Finland | 89.12 | 94.50 | 97.46 | 98.18 |
| France | 88.91 | 94.27 | 97.72 | 98.69 |
| Gabon | 15.89 | 17.14 | 23.59 | 32.41 |
| Gambia | 15.14 | 16.31 | 19.45 | 24.66 |
| Georgia | 61.16 | 67.17 | 63.76 | 66.87 |
| Germany | 91.08 | 96.35 | 98.16 | 98.55 |
| Ghana | 19.68 | 23.27 | 27.83 | 36.01 |
| Greece | 92.29 | 95.67 | 97.77 | 97.57 |
| Greenland | 48.67 | 55.68 | 58.98 | 65.71 |
| Grenada | 40.30 | 50.56 | 53.93 | 58.47 |
| Guam | 35.40 | 41.41 | 34.41 | 34.30 |
| Guatemala | 18.73 | 32.21 | 40.93 | 44.82 |
| Guinea | 8.06 | 8.83 | 11.79 | 14.96 |
| Guinea-Bissau | 8.01 | 8.54 | 12.83 | 16.18 |
| Guyana | 26.36 | 34.83 | 35.22 | 42.38 |
| Haiti | 8.05 | 11.14 | 13.21 | 19.21 |
| Honduras | 24.13 | 33.15 | 40.32 | 45.62 |
| Hungary | 72.86 | 83.39 | 86.46 | 89.90 |
| Iceland | 93.72 | 97.32 | 98.43 | 98.77 |
| India | 17.10 | 22.83 | 33.01 | 43.18 |
| Indonesia | 9.03 | 9.99 | 13.70 | 18.23 |
| Iran (Islamic Republic of) | 43.05 | 59.22 | 64.83 | 75.98 |
| Iraq | 35.67 | 37.46 | 52.51 | 63.76 |
| Ireland | 88.21 | 93.52 | 97.84 | 98.77 |
| Israel | 81.31 | 88.61 | 93.15 | 94.80 |
| Italy | 91.89 | 96.50 | 98.34 | 98.87 |
| Jamaica | 49.54 | 56.16 | 62.17 | 62.12 |
| Japan | 90.60 | 95.04 | 97.95 | 98.60 |
| Jordan | 43.03 | 54.45 | 68.35 | 77.69 |
| Kazakhstan | 50.04 | 44.47 | 58.27 | 72.53 |
| Kenya | 22.49 | 23.32 | 23.20 | 32.00 |
| Kiribati | 3.43 | 3.96 | 4.71 | 5.76 |
| Kuwait | 70.97 | 77.03 | 82.74 | 88.81 |
| Kyrgyzstan | 43.87 | 42.86 | 52.10 | 61.87 |
| Lao People's Democratic Republic | 1.26 | 2.18 | 6.10 | 10.88 |
| Latvia | 73.84 | 77.87 | 84.86 | 89.83 |
| Lebanon | 50.78 | 67.49 | 82.58 | 89.22 |
| Lesotho | 13.81 | 10.11 | 10.96 | 17.25 |
| Liberia | 11.04 | 13.72 | 21.99 | 24.21 |
| Libya | 47.19 | 56.06 | 70.86 | 69.74 |
| Lithuania | 76.58 | 82.23 | 84.54 | 88.20 |
| Luxembourg | 88.65 | 95.65 | 98.32 | 98.78 |
| Madagascar | 10.91 | 12.22 | 15.04 | 18.44 |
| Malawi | 13.26 | 13.58 | 19.27 | 24.11 |
| Malaysia | 18.67 | 23.93 | 32.14 | 40.20 |
| Maldives | 11.96 | 20.84 | 38.54 | 47.52 |
| Mali | 10.13 | 14.42 | 18.81 | 22.94 |
| Malta | 86.41 | 92.08 | 95.57 | 97.26 |
| Marshall Islands | 6.53 | 4.59 | 6.47 | 9.03 |
| Mauritania | 12.89 | 18.34 | 24.18 | 34.47 |
| Mauritius | 33.70 | 38.86 | 39.01 | 44.53 |
| Mexico | 39.55 | 55.72 | 61.78 | 66.86 |
| Micronesia (Federated States of) | 6.15 | 6.80 | 9.73 | 13.48 |
| Monaco | 95.72 | 97.58 | 98.75 | 99.12 |
| Mongolia | 24.97 | 24.75 | 42.37 | 50.66 |
| Montenegro | 80.73 | 79.81 | 86.03 | 89.52 |
| Morocco | 26.27 | 34.20 | 43.64 | 55.08 |
| Mozambique | 5.71 | 9.21 | 12.78 | 18.24 |
| Myanmar | 3.79 | 4.55 | 8.87 | 16.34 |
| Namibia | 14.76 | 16.65 | 26.19 | 39.42 |
| Nauru | 17.72 | 12.48 | 12.43 | 22.02 |
| Nepal | 10.86 | 18.67 | 27.03 | 36.16 |
| Netherlands | 94.01 | 96.17 | 98.80 | 99.25 |
| New Zealand | 94.99 | 97.65 | 98.52 | 99.13 |
| Nicaragua | 33.72 | 39.36 | 54.00 | 64.00 |
| Niger | 8.08 | 9.66 | 15.08 | 17.97 |
| Nigeria | 14.21 | 16.42 | 26.02 | 29.41 |
| Niue | 24.75 | 27.58 | 33.25 | 36.15 |
| North Macedonia | 56.26 | 67.64 | 77.40 | 81.26 |
| Northern Mariana Islands | 35.77 | 41.39 | 38.41 | 39.62 |
| Norway | 90.61 | 93.96 | 96.56 | 97.33 |
| Oman | 46.57 | 67.54 | 75.45 | 81.74 |
| Pakistan | 13.50 | 14.49 | 22.37 | 31.75 |
| Palau | 29.33 | 31.74 | 33.96 | 36.40 |
| Palestine | 44.12 | 48.90 | 54.22 | 65.95 |
| Panama | 48.79 | 61.63 | 65.71 | 73.74 |
| Papua New Guinea | 4.83 | 4.82 | 4.68 | 6.34 |
| Paraguay | 37.11 | 43.73 | 49.20 | 60.18 |
| Peru | 27.14 | 41.67 | 53.93 | 65.74 |
| Philippines | 16.20 | 15.33 | 16.73 | 20.68 |
| Poland | 52.04 | 66.31 | 75.01 | 80.42 |
| Portugal | 74.25 | 86.32 | 92.66 | 94.79 |
| Puerto Rico | 68.57 | 77.93 | 85.49 | 87.66 |
| Qatar | 45.85 | 59.56 | 78.66 | 83.69 |
| Republic of Korea | 64.11 | 84.11 | 94.25 | 96.48 |
| Republic of Moldova | 61.05 | 66.44 | 70.13 | 79.53 |
| Romania | 60.90 | 72.74 | 81.18 | 85.87 |
| Russian Federation | 70.73 | 68.82 | 81.20 | 87.54 |
| Rwanda | 6.56 | 6.77 | 19.26 | 28.63 |
| Saint Kitts and Nevis | 54.20 | 64.03 | 68.50 | 70.19 |
| Saint Lucia | 43.34 | 54.39 | 62.52 | 63.81 |
| Saint Vincent and the Grenadines | 47.42 | 52.15 | 56.96 | 57.77 |
| Samoa | 12.62 | 14.67 | 15.26 | 17.67 |
| San Marino | 93.48 | 96.76 | 98.11 | 98.59 |
| Sao Tome and Principe | 18.19 | 22.29 | 30.71 | 38.97 |
| Saudi Arabia | 35.19 | 50.30 | 68.64 | 81.63 |
| Senegal | 13.13 | 14.60 | 19.06 | 24.31 |
| Serbia | 61.26 | 71.39 | 81.79 | 86.11 |
| Seychelles | 22.17 | 24.70 | 31.01 | 36.85 |
| Sierra Leone | 11.88 | 12.30 | 15.96 | 22.44 |
| Singapore | 79.91 | 87.99 | 94.58 | 96.28 |
| Slovakia | 78.75 | 85.52 | 90.55 | 93.77 |
| Slovenia | 82.54 | 87.52 | 93.24 | 95.87 |
| Solomon Islands | 6.30 | 7.36 | 6.88 | 11.27 |
| Somalia | 4.36 | 2.64 | 3.88 | 5.93 |
| South Africa | 29.02 | 25.19 | 31.45 | 41.20 |
| South Sudan | 8.28 | 8.05 | 10.54 | 12.76 |
| Spain | 90.39 | 95.50 | 98.06 | 98.84 |
| Sri Lanka | 24.37 | 26.53 | 37.26 | 49.17 |
| Sudan | 17.76 | 24.46 | 31.40 | 43.22 |
| Suriname | 32.56 | 35.34 | 42.73 | 48.59 |
| Sweden | 91.42 | 95.20 | 96.46 | 97.23 |
| Switzerland | 93.59 | 97.46 | 98.62 | 98.98 |
| Syrian Arab Republic | 37.61 | 52.22 | 66.21 | 70.07 |
| Taiwan (Province of China) | 43.16 | 50.05 | 57.21 | 59.77 |
| Tajikistan | 32.62 | 31.81 | 35.53 | 40.36 |
| Thailand | 22.18 | 30.48 | 42.01 | 50.00 |
| Timor-Leste | 3.53 | 5.05 | 9.01 | 13.05 |
| Togo | 17.19 | 14.56 | 19.24 | 26.35 |
| Tokelau | 13.34 | 15.73 | 20.69 | 26.22 |
| Tonga | 14.37 | 14.26 | 14.55 | 17.84 |
| Trinidad and Tobago | 43.02 | 49.30 | 58.59 | 61.26 |
| Tunisia | 49.91 | 62.63 | 72.66 | 81.40 |
| Turkey | 34.07 | 49.03 | 70.74 | 79.28 |
| Turkmenistan | 39.91 | 44.57 | 52.20 | 61.35 |
| Tuvalu | 8.80 | 11.12 | 13.27 | 16.11 |
| Uganda | 11.52 | 12.88 | 19.87 | 26.72 |
| Ukraine | 64.66 | 65.45 | 76.84 | 78.46 |
| United Arab Emirates | 38.24 | 48.03 | 52.36 | 62.24 |
| United Kingdom | 89.54 | 94.05 | 96.97 | 97.44 |
| United Republic of Tanzania | 14.91 | 16.23 | 22.08 | 27.80 |
| United States of America | 94.36 | 97.10 | 97.92 | 97.72 |
| United States Virgin Islands | 50.35 | 60.06 | 65.18 | 65.19 |
| Uruguay | 61.57 | 71.13 | 75.71 | 79.10 |
| Uzbekistan | 45.19 | 45.41 | 51.95 | 59.59 |
| Vanuatu | 5.80 | 5.95 | 5.38 | 6.83 |
| Venezuela (Bolivarian Republic of) | 41.39 | 57.62 | 66.44 | 68.78 |
| Viet Nam | 13.55 | 17.79 | 27.44 | 38.00 |
| Yemen | 17.09 | 21.94 | 31.31 | 35.69 |
| Zambia | 10.51 | 9.31 | 16.63 | 29.85 |
| Zimbabwe | 27.01 | 25.75 | 16.87 | 23.62 |

* QCI = quality of care index
